# Correction: Conflicting ‘mother-scientist’ roles. An innovative application of basket analysis in social research

**DOI:** 10.1371/journal.pone.0303721

**Published:** 2024-05-09

**Authors:** Ewa Krause, Renata Tomaszewska, Aleksandra Pawlicka

There are errors in the Author Contributions. The correct contributions are: [1]

**Conceptualization:** Ewa Krause, Aleksandra Pawlicka, Renata Tomaszewska.**Data curation:** Ewa Krause, Aleksandra Pawlicka.**Formal analysis:** Ewa Krause, Aleksandra Pawlicka, Renata Tomaszewska.**Funding acquisition:** Ewa Krause, Renata Tomaszewska.

**Investigation:** Ewa Krause.

**Software:** Aleksandra Pawlicka.**Supervision:** Renata Tomaszewska.**Writing–original draft:** Ewa Krause, Aleksandra Pawlicka. Renata Tomaszewska**Writing–review & editing:** Ewa Krause, Aleksandra Pawlicka. Renata Tomaszewska.
