# Supplementary material for: Conflicting ’mother-scientist’ roles. An innovative application of basket analysis in social research
Source: PLoS One. 2022 Oct 18;17(10):e0276201. doi: 10.1371/journal.pone.0276201 (PMC9578633; doi:10.1371/journal.pone.0276201)
Supplement: Attachment — Submitted filename: 24.07_plos_one.Answers to the Reviewers.docx [file pone.0276201.s001.docx]

Answers to the Reviewers’ comments concerning:

PONE-D-21-40342R1

“Conflicting ’mother-scientist’ roles. An innovative application of basket analysis in social research.”

written by Ewa Krause, Renata Tomaszewska and Aleksandra Pawlicka

Dear Dr. Pawlicka,

Thank you for submitting your manuscript to PLOS ONE. After careful consideration, we feel that it has merit but does not fully meet PLOS ONE’s publication criteria as it currently stands. Therefore, we invite you to submit a revised version of the manuscript that addresses the points raised during the review process.
Your manuscript has been scientifically judged for a second time, finding out that our reviewer still raises (among others) several concerns over the technical background and data interpretation of the manuscript. Nwevertheless, and since I personally believe this paper has potential, IO would like to give you the chance to re-revise the paper, in order to reconsider the editorial decision.

Please submit your revised manuscript by Jul 29 2022 11:59PM. If you will need more time than this to complete your revisions, please reply to this message or contact the journal office at [plosone@plos.org](mailto:plosone@plos.org). When you're ready to submit your revision, log on to <https://www.editorialmanager.com/pone/> and select the 'Submissions Needing Revision' folder to locate your manuscript file.

Please include the following items when submitting your revised manuscript:

- A rebuttal letter that responds to each point raised by the academic editor and reviewer(s). You should upload this letter as a separate file labeled 'Response to Reviewers'.
- A marked-up copy of your manuscript that highlights changes made to the original version. You should upload this as a separate file labeled 'Revised Manuscript with Track Changes'.
- An unmarked version of your revised paper without tracked changes. You should upload this as a separate file labeled 'Manuscript'.

If you would like to make changes to your financial disclosure, please include your updated statement in your cover letter. Guidelines for resubmitting your figure files are available below the reviewer comments at the end of this letter.

If applicable, we recommend that you deposit your laboratory protocols in [protocols.io](http://protocols.io) to enhance the reproducibility of your results. Protocols.io assigns your protocol its own identifier (DOI) so that it can be cited independently in the future. For instructions see: <https://journals.plos.org/plosone/s/submission-guidelines#loc-laboratory-protocols>. Additionally, PLOS ONE offers an option for publishing peer-reviewed Lab Protocol articles, which describe protocols hosted on [protocols.io](http://protocols.io). Read more information on sharing protocols at <https://plos.org/protocols?utm_medium=editorial-email&utm_source=authorletters&utm_campaign=protocols>.

We look forward to receiving your revised manuscript.

Kind regards,

Sergio A. Useche, Ph.D.
Academic Editor
PLOS ONE

Dear Professor Useche,

Thank you for reading our paper and for all the feedback you’ve given us. We would like to thank you for your positive approach towards our paper, it made us really happy that you found our work interesting and worth publishing. In this revision, we have implemented the suggested alterations.

**Comments to the Author**

Reviewer #1: Thank you for submitting the revised copy of the manuscript; I do find the manuscript very interesting and the topic worth being published; However, the manuscript suffers from significant issues and I believe it can not be accepted in this format:

Thank you for your analysis of our paper and for your time used on the review! We’ve implemented the improvements as well as we can.

Still, the theoretical framework is unclear; I suggest having a separate section for this and supporting it with a diagram presenting the different variables.
I have serious concerns about the study validity and reliability assessment and analysis; please clarify them in a separate section how you did test the questionnaire validity and reliability in the method section.

Thank you for these remarks!

The questions of validity and reliability have now been answered in a separate subsection on page 21, by remarking e.g.: The relevance and reliability of the questionnaire administered to the surveyed female scientists was optimized by conducting a pilot study (traditional version: February - April 2019; online: November - December 2019). The actual study was conducted in January, February and March 2020.

We’ve also made it clearer that “The experiment was conducted according to the proper methodology, taking into account the control, replication and sample size.”

Still, there are no cites for studies that consider the context of this manuscript, especially the theoretical framework.

Thank you for this suggestion. To make the theoretical framework stand out, we’ve now emphasized it on Page 8. We’ve also added the following paragraph on the conflict along with the references to the studies, on Pages 8-9, to make the theoretical framework clearer:

The problem of the conflict between roles emerged more than 50 years ago (8). Back then, it was observed that the role demands of participation in a particular organization conflict with the need to participate in other social groups (9)(10). Based on this observation, inconsistent pressures within the family and work, mainly affecting women, were identified. It was found that women who are trying to meet the demands of both roles are subject to constant tension, as the need to perform one role is an obstacle to the effective performance of the other. Reconciling work and family responsibilities is a major challenge for many women and is often a source of conflict, which takes two forms: 1) work versus family (W-F), when the demands of the work role impede the fulfilment of the primary roles in the family, i.e., wife/partner and mother; 2) family versus work (F-W), when family responsibilities impede the fulfilment of the work role (11)(12).

There is also a new illustration, showing the foundation of the theoretical framework. It is to be found on page 5.

As for the second part of the experiment, we’ve now made it stand out ore that although we applied the basket analysis in a new, innovative way (social studies, and precisely – the analysis of the social role conflict), we’ve strictly followed the methodology, on Page 31:

“Having gathered, analysed and interpreted the data from the study, the authors went on to apply the basket analysis principles to it, and analyse it according to the methodology.”

We’ve also provided references for other studies using the basket analysis, on Page 32.

You did have the part on the mechanisms behind delays in women's scientific career development; please have it as a separate section and expand on it supported with references.

Thank you for this valuable input! We’ve made this onto a separate section and supported the points with the references, on Pages 16-17.

The discussion suffers from citing other studies to distinguish between opinion and empirical evidence; the authors did not elaborate enough on this point.
The discussion did not discuss the results raised by each research question presented in the study.

Thank you for pointing this out. We’re aware that with the pioneering nature of our paper, i.e., very limited number of studies on women scientists and no other application of basket analysis in the matter, there are not many references we’re able to provide to emphasize the empirical evidence rather than opinions. We’ve now added this remark in the Threats to Validity section on Page 39.

We’ve now moved the answers to the research questions to the new, completely rebuilt Conclusions section, to make them stand out more:

**Conclusions**

This paper has discussed the selected part of a larger study of mothers-scientists, which concentrated on the conflict of roles and its dimensions.

In the course of the study described in this paper, the answers to all the Research Questions were found:

- The answer to the research question 1.: Do the surveyed women employed at academic institutions in Poland experience conflict between scientific career and motherhood? If so, what kind of conflict?

As it was uncovered, most of the respondents declare they experience the conflict between the roles of a mother and a scientist, to a varying degree. The intensity of the perceived conflict tends to be related to the scientific degree the woman holds – the lower the degree, the more likely she is to experience severe conflict.

- The answer to research question 2.: What are the dimensions of the conflict between academic career and motherhood, according to the surveyed women employed at academic institutions in Poland?

With regard to the dimensions of the mother-researcher role conflict that the respondents currently concern or concerned in the past, the most frequently declared dimension is the time-related one, then subsequently the emotional dimension, and lastly the financial dimension. A substantial number of female scientists declare that they experience more than one dimension of conflict.

- Research question 3.: Is the basket analysis method able to find possible correlations between the factors characterising the respondents and the types and dimensions of the conflict between academic career and motherhood they claim to experience?

The results of the study were then analysed using the basket analysis tool – the apriori algorithm - which objectively confirmed the occurrence of correlations between the factors characterising the respondents and the types and dimensions of conflict occurring between scientific career and motherhood declared by. This confirms the usefulness of the tool beyond its typical, traditional use in business - as in this case, in the study of a large social group.

I highly recommend the authors get assistance from an English professional writer since there are a lot of type and grammar mistakes and unclear sentences.

Thank you for pointing this out! The paper has been proofread by a linguist and the typos/ grammar mistakes have been corrected.

The structure of the paper needs a lot of work so the manuscript can sound publishable.
I placed comments on the manuscript; Please refer to it as well and response in a table.
If the authors would like to apply the changes: please place the comments (above and in the manuscript) and the response in a table.
Having the comments and the response been made with a page number it will be much easier to follow.

Thank you! We’ve attached the track changes document, along with the clean version and this letter. We’ve mentioned the page numbers whenever relevant.

[NOTE: If reviewer comments were submitted as an attachment file, they will be attached to this email and accessible via the submission site. Please log into your account, locate the manuscript record, and check for the action link "View Attachments". If this link does not appear, there are no attachment files.]

The link did not appear.

Once again, thank you for your positive approach, thorough analysis of our paper and the following insightful comments. We have addressed and implemented all your suggestions to significantly increase the quality of our paper.

Allow us to acknowledge, recognize and appreciate your positive approach and your sharp scientific acumen. Having addressed and implemented all you suggestions, we believe this paper is now fit for publication in PLOS One.

Sincerely,

The Authors
