# Supplementary material for: Conflicting ’mother-scientist’ roles. An innovative application of basket analysis in social research
Source: PLoS One. 2022 Oct 18;17(10):e0276201. doi: 10.1371/journal.pone.0276201 (PMC9578633; doi:10.1371/journal.pone.0276201)
Supplement: Attachment — Submitted filename: 21.09_plos_one.Answers to the Reviewers.docx [file pone.0276201.s002.docx]

Answers to the Reviewers’ comments concerning:

PONE-D-21-40342R2

Conflicting ’mother-scientist’ roles. An innovative application of basket analysis in social research.

PLOS ONE

written by Ewa Krause, Renata Tomaszewska and Aleksandra Pawlicka

Dear Dr. Pawlicka,

Thank you for submitting your manuscript to PLOS ONE. After careful consideration, we feel that it has merit but does not fully meet PLOS ONE’s publication criteria as it currently stands. Therefore, we invite you to submit a revised version of the manuscript that addresses the points raised during the review process.

Your paper has been re-reviewed. Overall, our referee suggests the consideration of your manuscript for acceptance, but only after a new phase of revisions, in which some minor (but still relevant) points must be addressed by you to the best of the possibilities. Please find the full set of comments below.

Please submit your revised manuscript by Nov 04 2022 11:59PM. If you will need more time than this to complete your revisions, please reply to this message or contact the journal office at [plosone@plos.org](mailto:plosone@plos.org). When you're ready to submit your revision, log on to <https://www.editorialmanager.com/pone/> and select the 'Submissions Needing Revision' folder to locate your manuscript file.

Please include the following items when submitting your revised manuscript:

- A rebuttal letter that responds to each point raised by the academic editor and reviewer(s). You should upload this letter as a separate file labeled 'Response to Reviewers'.
- A marked-up copy of your manuscript that highlights changes made to the original version. You should upload this as a separate file labeled 'Revised Manuscript with Track Changes'.
- An unmarked version of your revised paper without tracked changes. You should upload this as a separate file labeled 'Manuscript'.

If you would like to make changes to your financial disclosure, please include your updated statement in your cover letter. Guidelines for resubmitting your figure files are available below the reviewer comments at the end of this letter.

If applicable, we recommend that you deposit your laboratory protocols in [protocols.io](http://protocols.io) to enhance the reproducibility of your results. Protocols.io assigns your protocol its own identifier (DOI) so that it can be cited independently in the future. For instructions see: <https://journals.plos.org/plosone/s/submission-guidelines#loc-laboratory-protocols>. Additionally, PLOS ONE offers an option for publishing peer-reviewed Lab Protocol articles, which describe protocols hosted on [protocols.io](http://protocols.io). Read more information on sharing protocols at <https://plos.org/protocols?utm_medium=editorial-email&utm_source=authorletters&utm_campaign=protocols>.

We look forward to receiving your revised manuscript.

Kind regards,

Sergio A. Useche, Ph.D.
Academic Editor
PLOS ONE

Dear Professor Useche,

Thank you for reading our paper and for all the heart-warming words! We would like to thank you for your finding our work interesting and worth publishing. We’ve worked very hard all day today and in this revision, we have implemented the suggested alterations.

6. Review Comments to the Author

Reviewer #2: Dear Authors,
First of all, thanks for contributing with this interesting research about “Conflicts mother’s scientist’s role “After a careful assessment of the paper, I believe the reviewed manuscript addresses a pertinent research problem (i.e. female scientists and motherhood) for being considered as publishable in PLOS ONE. Of course, some points need to be addressed beforehand.

Dear Reviewer, thank you so much for this very positive review and absolutely lovely words! It was a real pleasure to get this kind of feedback. We’ve worked hard all day and we have addressed and implemented all your suggestions.

In general, the structure of the paper is adequate; however, the presentation can be clearer and more concise. In this regard, some concerns, queries and suggestions raised during my review must be addressed, in order to optimize the manuscript contents and its suitability for the journal:

Thank you for your feedback! We’ve addressed all the comments, namely:

• ABSTRACT: The relation between these concepts is not clear:
Please introduce the results in the abstract

To clarify the concepts and include the results in the abstract, we’ve added the following passage:

“The paper shows that, according to the study results, most respondents declare they indeed experience the conflict between the roles of a mother and a scientist. The most frequently declared dimension of the conflict is the time-related one, then subsequently the emotional dimension, and lastly the financial dimension; many scientists declare they experience more than one dimension of conflict. Lastly, the basket analysis tool objectively confirmed the occurrence of correlations between the factors characterising the respondents and the types and dimensions of conflict occurring between scientific career and motherhood.”

• METHODS: For a better understanding of the study, please include a section where each of the methods used can be clearly described.
It would be advisable to describe the process for answer the questions better for the sample

Thank you for this remark!

In the “Materials and methods” section, on pages 20-22, we’ve explained all the methods applied in the course in the study, namely:

“Besides the basket analysis, the following methods have also been employed in the course of the experiments presented in this paper:

- Method of critical analysis of sources (literature).

This method allows one to establish a list of the most important items of specialized or methodological literature related to a large extent to the problem. It is a common method and is applicable to all fields of scientific inquiry. Its essence is to demonstrate the expediency, originality and new treatment of the problem that has emerged and undertaken for research. It is necessary to demonstrate by this method what we know and what we do not know, what already exists and is contained (known) in the literature, and what does not exist and needs to be known and proven by research. Cognition is carried out by means of analysis and criticism of the literature of the subject (issue) under study. This method involves studying the news recorded in studies, documents and other communications. The selection of the sample, that is, the fragments of the messages analyzed, depends on what is the unit of analysis. These can be, for example, specific events, characters, individual words, definitions, expressions and others. Content analysis is carried out in three stages: coding of the material (both explicit and implicit content), counting the coded by category of content, comparative analysis of the collected empirical material.

- Formal-legal document analysis method

This method is widely spread in the social sciences, it is applicable where the study takes into account the most diverse documents. The documents to be studied can be, therefore, any human products (material objects) that express a thought, vision, mission, achievement, proposal and serve to reproduce the actual activity or state of the organizational structure under study in the form of a legally or even customary document. It is, therefore, an extremely important, extensive, detailed and specific source material (for example, terms of reference) concerning a particular institution or person. In general, a distinction is made between classical (qualitative or descriptive) and modern (quantitative) document analysis. Classical document analysis is mainly based on their historical and literary interpretation. It is a search for individual characteristics specific to the analyzed document and its creator. Modern document analysis is an attempt to overcome the subjective nature of traditional (classical) analysis. It consists primarily of quantitative description and analysis of documents, with such description and analysis not limited to the use of absolute numbers or percentages. Great importance is attached to the accurate determination of the cognitive value of documents, including, in particular, the confirmation of their reliability and authenticity, that is, it is sought to demonstrate that the documents included in the analysis can be a legitimate basis for solving the problem of interest to the researcher, and that the time of their creation, author or creator and place of origin are well known to them. To each of these document analysis, different varieties of documents are subordinated. Content analysis involves the interpretation of the contents contained in the documents. Formal analysis of documents, on the other hand, is concerned with the external description of their appearance, the way they were drawn up, the degree of permanence or relevance with the intention that guided or was intended in the course of their creation.

- Diagnostic survey method

This method is sometimes - for the purposes of pedagogical research - called a diagnostic survey, representative group survey, opinion survey or simply a survey. In the most general terms, it is understood as a method of research, the primary function of which is to collect information about the problems of interest to the researcher as a result of verbal reports of the subjects, called respondents. The constructive feature of the survey method is "questioning," that is, probing opinions. In the case of written responses, it takes the form of surveys, and in the case of oral responses, it takes the form of interviews.

- Statistical methods

These are ways of quantitatively describing mass phenomena and the relationships between them, as well as presenting the results in a conventional form (tables, graphs, average values, correlation). From the results obtained, conclusions are derived on the basis of probability theory.”

The proper references have also been provided.

• CONCLUSION: Adequate, and within the scope of the data. However, study limitations could be improved.
Thank you for this suggestion! We’ve reconstructed a major part of the Threats to validity / study limitations section on page 46 as follows:

“**Threats to validity and study limitations**

The study came with a number of factors which might be considered to be limitations to it. The most significant factor were the criteria for the selection of respondents for the study. They can be considered as limitations of this study, as it only considered the respondents of one gender – i.e. women only. All the respondents belonged to the same professional group and had specific job titles/degrees/academic titles (M.Sc., Ph;). Moreover, all the respondents have been employed at a position which obliges to conduct research and development activity at an academic institution in Poland (excluding teaching positions, which do not oblige to conduct research and development work). All the respondents were employed at the same type of workplace, i.e. academic universities. Lastly, the study only concerned the respondents who were willing to take part in the research. The authors of this work are aware of those possible limitations of the study and will try to compare the results of the respondents belonging to other groups, such as scientist who play the role of a father. The authors would also like to conduct a similar study in other European countries, as it could be beneficial to check and compare if the situation of female scientists is similar there, or not.”

I congratulate you for your perseverance and effort made to carry out this work

Thank you so much for this heartwarming feedback! Your words have made our day!

Dear Reviewer, once again, thank you for your positive approach, thorough analysis of our paper and the following insightful comments. We have addressed and implemented all your suggestions to significantly increase the quality of our paper.

Having addressed and implemented all you suggestions, we believe this paper is now fit for publication in PLOS One.

Sincerely,

The Authors
